# Plans, hopes and ideas for mental health

**DOI:** 10.1192/pb.bp.116-053793

**Published:** 2017-02

**Authors:** John R. Ashton

**Affiliations:** 1 The UK's Faculty of Public Health, London

## Abstract

Mental health and the failings of the mental health services are in the spotlight as never before. Nowhere is this more apparent than in the often dire situation with regard to child and adolescent mental health. At the same time, there is a renewed interest in the scope for prevention of mental illness and distress, and in population approaches to mental well-being. It may come as a surprise to some that others have given such serious consideration to strategic approaches to public mental health as long ago as the 1950s. It appears that such consideration was squeezed out by the dominant concerns of serious and enduring mental illness and a prevailing biological view of psychiatry. The time is right to engage with this agenda in recognition of the importance of public mental health, not only for the individual and for families, but also for society as a whole and for the economy. The publication of a review of the subject by the Faculty of Public Health and the Mental Health Foundation is to be commended. Let us make sure it leads to action.

The recent announcement by UK Prime Minister David Cameron of a new initiative for mental health, with a particular emphasis on parenting classes,^1^ is most welcome. It comes at the end of a year in which there has been an increasing concern for the state of the nation's mental health, with a flurry of documents and reports, a campaign led by *The Times* newspaper^2^ and an increasing demand for parity of resourcing between mental and physical health. Interestingly, some of the pressure to do something specifically about child and adolescent mental health is coming from the independent schools sector. The schools have been expressing increasing concern about the mental well-being of the young people in their care, faced by an apparently steady increase in the incidence of distress manifested by levels of general anxiety and depression, and specifically the levels of eating disorder, self-harm and other behavioural manifestations. Not for the first time in public health, something that has long been a problem for the most disadvantaged in society is being taken seriously once it becomes an issue for the privileged. Nor should we ignore the opportunity presented for progress by the mobilisation of enlightened self-interest by those in positions of power and influence. After all, in Victorian times, the fact that cholera knew no social boundaries led to sanitary reform which was of benefit to rich and poor alike. More recently, once it became apparent that HIV/AIDS was not only a disease of stigmatised minorities, the research dollars rolled in.

As a public health physician who began his career as a psychiatrist and family doctor and is finishing as President of the UK's Faculty of Public Health, I find particular poignancy in returning to the theme of public mental health for my swansong year, a theme which I have chosen for the Faculty to focus on in 2015–2016. I appreciate and welcome this opportunity to share some thoughts with clinical colleagues in psychiatry based on 40 years of trying to make sense of some of the questions raised within a public health paradigm.

My journey from psychiatric registrar in Newcastle in the 1970s offers some perspective. As a student I was one of those medics whose interests spanned the humanities as well as the sciences. History and politics were always as interesting to me as biology, and when I came across the prospectus for the public health masters course at the London School of Hygiene & Tropical Medicine sometime in third year, it was clear to me that sooner or later I would be signing up. For the next 10 years I would religiously send for the latest edition. Fortunately, my interest was nurtured and kept alive by the remarkable social orientation of the Newcastle course, not just in family and community medicine but also in such mainstream clinical areas as paediatrics, psychiatry and obstetrics. The school was imbued with the spirit not just of the pioneering, community-oriented paediatrician Sir James Spence, but also that of Aberdonian obstetrician Dugald Baird through his Newcastle disciples. The strong social and community base was reinforced by a series of Deans of Medicine, who, while hard-nosed neurologists and endocrinologists themselves, supported the work of those such as Donald Irvine, who was centrally involved in establishing the country's first general practice training programme and later oversaw the General Medical Council. When I signed up for the psychiatric training rotation under the formidable Sir Martin Roth, amazingly comprehensive and intellectually stimulating as it turned out to be with placements in all aspects of mental health services, I found myself frustrated at the failure of those services to focus upstream to prevention and the promotion of mental health.

While as a registrar in the heady years of the challenges posed to orthodox practice by the likes of R.D. Laing and Thomas Szasz, I was exposed to the whole spectrum of ideas, from Freud and Jung to Kraepelin, Sargent and Eliot Slater. Although we had opportunities to cut our teeth on individual, group and marital therapy, the broader public health agenda remained elusive. I came to the conclusion that what was on offer was all too little and too late, and as soon as I had finished my training I moved into general practice in the hope of finding more fertile soil for prevention.

My next move took me to Southampton, where the pioneering dean of the new medical school, Donald Acheson, had created an exciting opportunity which seemed tailor-made for somebody like myself. In a university-run health centre in the local community, based on lines recommended by the celebrated Birmingham professor of public health Thomas McKeown, there were to be specialoid general practitioners – GP paediatricians, GP mediatricians (caring for grown-ups), GP geriatricians and a GP psychiatrist (me). Part of the time we would teach medical students, and the remainder was spent providing a combination of general practice, including out-of-hours services, and specialist expertise to the practice patients as well as supporting each other. As far as possible, we would look after the population of the Aldermoor estate (a public health notion), and consume our own smoke.

It was a stimulating time, but there were problems reconciling the competing claims of medical school and service as well as staffing issues. Southampton was within spitting distance of the London School of Hygiene & Tropical Medicine, so it was time to make the logical step into public health, and it was quite clear that I had made the right move. Validation came from, among others, John Wing and Julian Leff from the Maudsley, who also taught social psychiatry at the school, from visiting teachers from the London School of Economics (LSE), such as Bryan Abel Smith, who confirmed what students suspected, namely that ‘public health is the political wing of medicine’ and that ‘Parliament is the dispensary of public health’, and others that placed population health at the centre. It was one of those group learning experiences which stays with you down the ensuing years as a highlight and a transformational experience. Yet there was something missing.

In those days students on the public health masters courses at the School had the enormous privilege of a 2-year course, 1 year spent in the classroom and 1 year on a dissertation. The dissertation was a kind of blank cheque that enabled you to pursue something of special interest that would hopefully be built on in future years. And this is where my problem reasserted itself. What would be a suitable dissertation that majored on prevention and mental public health? I was already a member of the social psychiatry section of the Royal College of Psychiatrists and I took advice from as many people as I could find, including Sir Martin Roth. I drew a blank. The nearest anybody could get was early diagnosis and treatment in the community, what I now knew to be secondary prevention in public health, tertiary prevention being rehabilitation. Primary prevention was nowhere to be found.

And so in the end I hit on planned parenthood, something much better understood in a holistic sense in global health circles, and I carried out a series of studies into family planning and abortion at the population level of Wessex. In my subsequent career as a public health academic, as a regional and county director of public health, as an adviser to the World Health Organization on the Healthy Cities project, and most recently, as President of The UK's Faculty of Public Health, I have reconciled my angst that as a generalist with a population and environmental focus, all my work has ultimately to be judged by its impact on mental health and well-being. So what have I learned and what observations can I make faced with the promise that finally mental health is to be taken seriously?

One of the problems with mental health, as with physical health, is that the dominant approach is to work backwards, from a focus on treatment towards an interest in prevention. The exception is when there is an emergency, a disaster or a war, when needs must apply a public health population-based triage model if harm is to be minimised.

In the 1980s I attended a short course at the School for would-be volunteers to work in refugee camps in the Horn of Africa. One message stays with me almost 40 years later. If a small group of volunteers (doctors, nurses, engineers and so on) is deployed into a camp of 12 000 women and children in dire circumstances (the men are likely to be either already dead or off fighting somewhere), the first thing to do is not to start treating sick patients. Rather, it is to carry out a quick census of who is there and what skills they have, and to set about mobilising the expertise and supporting it.

This is not our traditional medical model, based as it is on putting up your plate outside a consulting room and offering services to those who can afford to pay, with no concern for the denominator of those with unmet need. Take the example of child and adolescent psychiatry. The large community surveys such as those on the Isle of Wight and in South London found that around 10% of children and adolescents suffer from such a level of emotional or conduct disorder as to require specialist help.^3^ In a borough of 500 000 population (about 70 000 children and adolescents), this will equate to about 7000 potential patients. In a fortunate district perhaps, optimistically, 1000 of those could be adequately managed by a typical child and adolescent mental health service (CAMHS). No district will ever have that kind of establishment. At the risk of being written off as a loony baby boomer, I would quote Mao Zedong: who is said to have claimed that ‘If the practice doesn't work, the theory is wrong’. We are starting at the wrong end of the telescope or focusing on the wrong part of the pyramid of needs. So what would public health say and what is to be done?

In 1961 Gerald Caplan published a book titled *An Approach to Community Mental Health*. Caplan was educated at Manchester medical school and worked at the Tavistock Institute in London and the Hadassah Centre in Jerusalem before moving to the USA, where his work was hugely influential, not least with the programme of community mental health centres under President Kennedy. I came across his book in the 1980s and have carried it round with me ever since.

Reading it again now, it is as relevant and fresh today as it was when it was written, and it is a mystery to me why it has not been a blueprint for how we have approached mental health during the intervening years. Perhaps it is because it includes a (very sensible) chapter on ego psychology, when British psychiatry has for so long been under the shadow of organic theorists and psychopharmacology? In essence, what Caplan proposes is a comprehensive community approach to preventive psychiatry and the provision of services which builds on individual and community assets including those of what he calls ‘caretaking agents’ and those in special positions in everyday life. He includes in this not just doctors and nurses but clergy, teachers, policemen and so on, and advocates a system built on up-skilling those in a position to play a protective and supportive role in everyday life as a first line.

Caplan describes administrative actions that can protect and support good mental health as well as personal and clinical interactions and redefines the role of those with specialist psychiatric expertise in building and supporting both capacity and capability for mental health and well-being. For me, using the example of child and adolescent mental health, this translates into a life cycle approach that starts with planned parenthood, builds on it with the Prime Minister's parenting classes, and ensures that all those in key interactions with parents and children have adequate skills to promote mental health and respond quickly to signs of distress. This extends to children themselves having the opportunity at school to develop mental resilience and skills for mutual mental health assistance with their peers. The administrative part includes key action on wider determinants of health such as economic and social security, housing and access to good educational and work opportunities. If all this is implemented, the question then arises as to what the formal system should be offering in primary care, building on recent developments in Improving Access to Psychological Therapies (IAPTS) and how serious breakdown and risk can be handled for the whole population of patients for whom this becomes necessary.^4^

One of the enemies of adopting this kind of comprehensive approach to mental health is the prevailing narrow and reductionist model of scientific evidence as illustrated by recent controversy over the concept of mental well-being as a researchable paradigm.^5^ For Caplan,

‘Our lack of knowledge in regard to the significance of the different factors has to be remedied by a continuation of existing research into aetiology. But, meanwhile preventive psychiatrists have been able to learn a lesson from public health colleagues in regard to handling of the problem of the multifactorial nature of the picture … The incidence of cases of clinical tuberculosis, for example, in any community is no longer conceived of in public health circles as being merely dependent upon the single factor of the presence or absence of the tubercle bacillus. It is recognised that there are many complicated issues that will determine whether a particular person exposed to the germ will contract the clinical disease: issues involving virulence of the germ, host susceptibility and various environmental factors’.^6^

In public health we have learned to take a whole-systems approach to whole and sub-populations and to use multiple interventions acting on the health ‘field’.

The list of factors of interest to those concerned with protecting and improving mental health, mental well-being and resilience, in addition to the proximal factors of those aspects of personal security already mentioned and the managed challenges that enable people to grow and thrive, includes a set of constructs such as locus of control, self-esteem and coherence. These can be difficult constructs to operationalise for research purposes, especially when they interact in complex systems, but tools can be developed, for example the Rosenberg Self-Esteem Scale,^7^ and in recent years mixed-methods and compound outcomes such as those used in Social Return on Investment^8^ have paved the way for practical interventions based on pragmatic considerations.

We must be careful to avoid the dangers of scientism. When John Snow took the handle from the Broad Street pump during the 1854 cholera epidemic in Soho, the cause of cholera was still believed to be the miasma. This was 20 years before Pasteur's ground-breaking research. And still nowadays a whole system of education based on the evidence-free assumption that team sports are character forming underpins the British public schools system.

Caplan's book concludes with a remarkably contemporary proposal for the development of comprehensive community psychiatry based on 11 concepts and assumptions that could well provide the starting point for a consideration as to how any new government funds might be committed. For myself, I have come to the conclusion that in addition to those things which government can and should do through ‘the pharmacy of public health’, there are three approaches, tried and tested in recent years, that should be regarded as delivery systems.

1. ‘Total place’ and ‘defined population’ as developed through Healthy Cities, Healthy Schools, Healthy Prisons and other settings.^9^
2. Asset-based community development as proposed by John McKnight and colleagues in Chicago.^10,11,12,13^ This approach maps and mobilises the gifts and talents of individuals, families and communities on the basis that:
  - they are half-full, not half-empty
  - it takes a village to raise a child
  - 90% of health and social care is lay care
  - unless professional practice supports self-efficacy it can be part of the problem rather than part of the solution.
3. Community-oriented primary (and secondary) care based on an epidemiological understanding of populations and responsibility for them, as practised by Sidney Kark and his colleagues over many years at the Hadassah Medical School in Jerusalem.^14^

In conclusion, I am optimistic that we have an opportunity to re-launch mental health in this country at the same time as developing parity and integration with physical health. The Faculty of Public Health is playing its part by launching a new public mental health report in June to share best practice among public health practitioners.^15^

There is a particular opportunity to pursue this approach in England, where NHS England's *Five Year Forward View*^16^ with its integrated ‘new care models’ is driving transformational change. However, the paradigm shift to a public health model with co-production at its heart is a precondition. More of the same just won't do.
